# Pain Management in Ambulatory Surgery—A Review

**DOI:** 10.3390/ph7080850

**Published:** 2014-07-24

**Authors:** Jan G. Jakobsson

**Affiliations:** Department of Anaesthesia & Intensive Care, Institution for Clinical Science, Karolinska Institutet, Danderyds Hospital, 182 88 Stockholm, Sweden; E-Mail: jan.jakobsson@ki.se

**Keywords:** ambulatory surgery, analgesia, multi-modal analgesia, balanced analgesia, postoperative pain

## Abstract

Day surgery, coming to and leaving the hospital on the same day as surgery as well as ambulatory surgery, leaving hospital within twenty-three hours is increasingly being adopted. There are several potential benefits associated with the avoidance of in-hospital care. Early discharge demands a rapid recovery and low incidence and intensity of surgery and anaesthesia related side-effects; such as pain, nausea and fatigue. Patients must be fit enough and symptom intensity so low that self-care is feasible in order to secure quality of care. Preventive multi-modal analgesia has become the gold standard. Administering paracetamol, NSIADs prior to start of surgery and decreasing the noxious influx by the use of local anaesthetics by peripheral block or infiltration in surgical field prior to incision and at wound closure in combination with intra-operative fast acting opioid analgesics, e.g., remifentanil, have become standard of care. Single preoperative 0.1 mg/kg dose dexamethasone has a combined action, anti-emetic and provides enhanced analgesia. Additional α-2-agonists and/or gabapentin or pregabalin may be used in addition to facilitate the pain management if patients are at risk for more pronounced pain. Paracetamol, NSAIDs and rescue oral opioid is the basic concept for self-care during the first 3–5 days after common day/ambulatory surgical procedures.

## 1. Introduction

Day or ambulatory surgery; coming and leaving the hospital the same day or within 23-hours after surgery is becoming increasingly adopted. There are several potential benefits associated with the avoidance of in-hospital care. The risk for hospital acquired infections and the potential negative effects of increased immobilization and subsequent risk for thrombo-embolic and anenergetic sequalae are also lowered. Enhanced recovery and shortened hospital stay has also been shown to reduce the risk for cognitive side effects.

**Figure 1:**
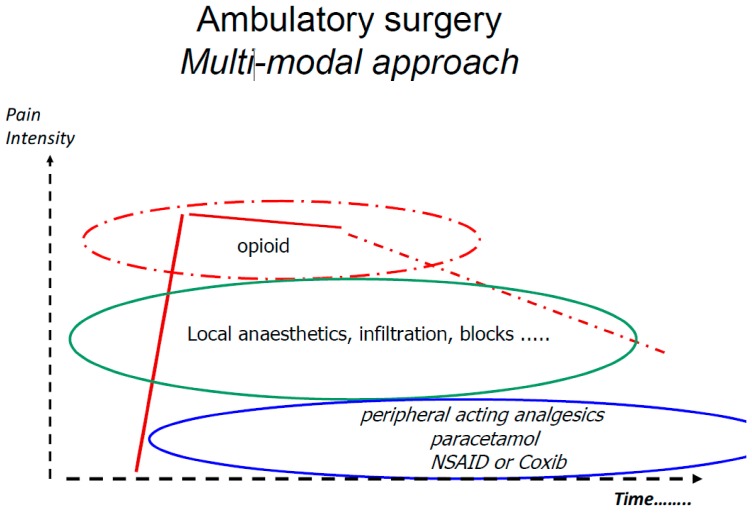
The basic principle for preventive multi-modal analgesia.

Early discharge demands a rapid recovery and low incidence and intensity of surgery and anaesthesia related side-effects; such as pain, nausea and fatigue. Patients must be fit enough and symptom intensity so low that self-care is a feasible in order to secure quality of care. Multi-modal or balanced analgesia [1] has become increasingly adopted as a opioid sparing pain management strategy. The multimodal approach was introduced to ambulatory surgery in the mid 1990ties more or less at the same time by the group in Helsinki (Finland) lead by Professor Kari Korrtila [2] and group in Toronto (Canada) under the lead of Professor Francis Chung [3]. In consists of combining analgesics with different mode of action and side-effect profile in order to achieve additive or preferentially synergistic analgesic effect but with a minimum of side effects, reducing the need for opioid analgesics and opioid related side effects. The basic concept includes analgesics such as paracetamol, NSAID/Coxibs and local anaesthetics (Figure 1), but there are numerous drug combinations, routes of administration and timing for administration that have been tested. The analgesic effects assessed as the number need to treat has been extensively analysed by the Bandolier team and is presented at their home page [4]. This paper aim at review the evidence-base for the concept of multi-modal analgesia for pain management following ambulatory surgery/anaesthesia.

## 2. Paracetamol

Paracetamol is one of most commonly used analgesics and antipyretics. Graham *et al.* [5] addressed the pharmacology. Paracetamol is, on average, a weaker analgesic than NSAIDs or COX-2 selective inhibitors but is often preferred because of its better tolerance. Despite the similarities to NSAIDs, the mode of action of paracetamol has been uncertain, but it is now generally accepted that it inhibits COX-1 and COX-2 through metabolism by the peroxidase function of these isoenzymes. Paracetamol, NSAIDs and selective COX-2 inhibitors all have central and peripheral effects. As is the case with the NSAIDs, including the selective COX-2 inhibitors, the analgesic effects of paracetamol are reduced by inhibitors of many endogenous neurotransmitter systems including serotonergic, opioid and cannabinoid systems. There is considerable debate about the hepatotoxicity of therapeutic doses of paracetamol. Much of the toxicity may result from overuse of combinations of paracetamol with opioids which are widely used, particularly in USA. It is available as over-the-counter medication in many countries. It is known to be associated with negative health effects when used in higher doses and or for prolonged time [6]. Recently FDA recommended dose limitation in drug combinations to 325 mg paracetamol [7]. There are today several different formulations of paracetamol. The dose effect for standard oral administration was shown by Qi *et al.* [8]. They found 1,000 mg paracetamol to provide clinically meaningful and statistically significantly greater efficacy in treating postsurgical dental pain compared with paracetamol 650 mg and placebo. Yue *et al.* [9] sought to investigate the dose-response efficacy and speed of onset of pain relief of a fast-dissolving paracetamol formulation compared with lower doses of paracetamol and placebo in dental patients after impacted third molar extraction. They found onset of first perceptible relief in subjects treated with FD-APAP 1,000 mg was 15 min, which was 32% and 25% significantly shorter than onset of pain relief of fast-dissolving paracetamol formulation 500 mg (22 min) and standard paracetamol 650 mg (20 min), respectively. Fast-dissolving paracetamol formulation 500 mg and the standard paracetamol 650 mg demonstrated efficacy over placebo for most of the measurements; however, their effects were significantly lower and lasted for a shorter period of time than for fast-dissolving paracetamol formulation 1,000 mg.

Holmer-Petterson *et al.* [10] have shown that immediate post-operative oral administration of paracetamol as part of multimodal pain management resulted in a huge and unpredictable variation in plasma concentration compared with intravenous administration. Brett *et al.* [11] found that only a minority of patients receiving the 1.0 g oral dose preoperatively had plasma levels in the therapeutic analgesic range. McNicol *et al.* [12] have made a meta-analysis around the effects of intravenous paracetamol. They found that patients receiving propacetamol or paracetamol required 30% less opioid over 4 h and 16% less opioid over 6 h than those receiving placebo. However, this did not translate into a reduction in opioid-induced adverse events (AEs). Apfel *et al.* [13] determined the effect IV paracetamol has on patient satisfaction, a pooled analysis from methodologically homogenous studies. The primary endpoint was “excellent” satisfaction and the secondary endpoint was “good” or “excellent” satisfaction at 24 h after first study drug administration. Patients receiving IV paracetamol were more than twice as likely as those who received placebo to report “excellent” patient satisfaction ratings (32.3% *vs.* 15.9%, respectively). Of all variables that remained statistically significant in the multivariable analysis (*i.e.*, type of surgery, duration of anaesthesia, last pain rating, and opioid consumption), IV paracetamol had the strongest positive effect on “excellent” patient satisfaction with an odds ratio of 2.76 (95% CI 1.81–4.23). Results for “excellent” or “good” satisfaction were similar. When given as part of a perioperative analgesic regimen, IV paracetamol was associated with significantly improved patient satisfaction. Bailey *et al.* [14] conducted a meta-analysis around ibuprofen and/or paracetamol (acetaminophen) for pain relief after surgical removal of lower wisdom teeth. They found in a total of 2,241 participants were enrolled in these trials. Ibuprofen was found to be a superior analgesic to paracetamol at several doses with high quality evidence suggesting that ibuprofen 400 mg is superior to 1,000 mg paracetamol based on pain relief (estimated from TOTPAR data) and the use of rescue medication meta-analyses. They concluded that ibuprofen is superior to paracetamol at doses of 200 mg to 512 mg and 600 mg to 1,000 mg respectively based on pain relief and use of rescue medication data collected at six hours postoperatively. The majority of this evidence (five out of six trials) compared ibuprofen 400 mg with paracetamol 1,000 mg, these are the most frequently prescribed doses in clinical practice.

## 3. NSAIDS and Coxibs

The Coxibs have become an interesting option in postoperative pain management. The less pronounced effect on platelet function and subsequent lower risk for impaired hemeostasis makes them, in theory, a preferred option to the non-selective traditional NSAIDs. The benefit *versus* risk for a more generalized use of Coxibs must, however, be based on a thorough evaluation of the overall benefits and risks for the use of NSAIDs and a further evaluation on whether the specific therapeutic features of the Coxibs provide benefits outweighing their increased cost [15].

Kawano *et al.* [16] investigated the effects of postoperative analgesia with ketoprofen on cognitive functions in aged animals and compared its effectiveness to morphine. Rats were randomly allocated to one of four groups: isoflurane anesthesia without surgery (group C), isoflurane anesthesia with laparotomy (group IL), and isoflurane anesthesia with laparotomy plus postoperative analgesia with ketoprofen or morphine. There was no difference in postoperative locomotor activity among groups. In group IL, postoperative pain levels assessed by the Rat Grimace Scale significantly increased until 8 h after surgery, which was similarly inhibited by both ketoprofen and morphine. Cognitive function was assessed using radial arm maze testing for 12 consecutive days from postoperative day 3. Cai *et al.* [17] conducted a study in rabbits, and found that a 7-day regimen of appropriate doses of diclofenac sodium and parecoxib did not adversely affect osseointegration of dental implants and bone healing in calvaria, neither short nor long term (12 weeks). The meta-analysis by Clarke *et al.* [18] published in 2012 around Single dose oral etoricoxib for acute postoperative pain in adults showed that at least 50% pain relief was reported by 66% with etoricoxib 120 mg and 12% with placebo (NNT 1.8 (1.7 to 2.0)). For dental studies only the NNT was 1.6 (1.5 to 1.8). Although the new study almost doubled the number of participants in included studies it added only about 25% more data for the 120 mg dose and the result was unchanged. Other doses (60, 90, 180, and 240 mg) were each studied in only one treatment arm and we did not undertake pooled analysis. Significantly fewer participants used rescue medication over 24 h when taking etoricoxib 120 mg than placebo (NNT to prevent remedication 2.2 (1.9 to 2.8)), and the median time to use of rescue medication was 20 h for etoricoxib and two hours for placebo. Adverse events were reported at a similar rate to placebo, with no serious events.

The potential negative effects, risk for myocardial infarct, thromboembolic and cardiovascular events associated to the long-term use of NSAIDs and Coxibs most of course be considered [19]. The risk *vs.* benefit during short term postoperative use seems reasonably positive. Whether the more selective COX-II-inhibitors provide clinically important benefits, reduced effect of platelet and lower risk for GI-irritation is still a matter of discussion [20].

## 4. Combination NSAID and Paracetamol

There is an increasing amount of data supporting the combination of paracetamol and an NSAID. There is a recent meta analysis by Derry *et al.* [21] showing that ibuprofen plus paracetamol combinations provided better analgesia than either drug alone (at the same dose), with a smaller chance of needing additional analgesia over about eight hours, and with a smaller chance of experiencing an adverse event.

## 5. Local Anaesthesia

**Figure 2:**
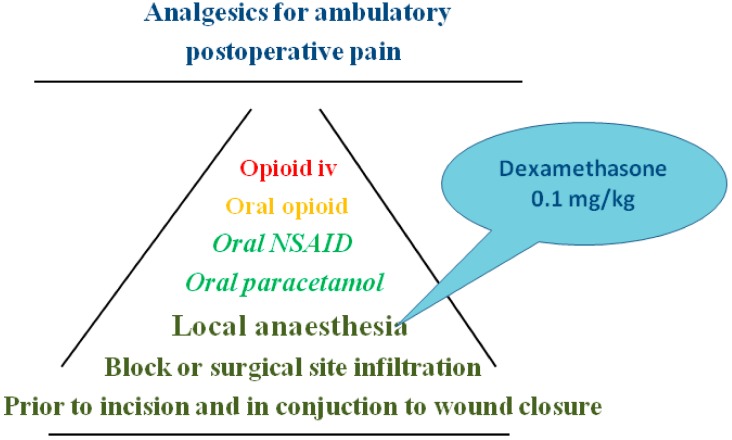
Multi-modal analgesia; the basic and escalating addition of drugs.

Local anaesthesia has been used in conjunction to ambulatory surgery since long and was part of the initial successful studies by the Helsinki and Chung groups. It may be administered at the surgical site or as nerve block. Local anaesthesia provided prior to start of surgery is a most fundamental part of the multi-modal analgesia strategy see Figure 2.

Ultrasound guided nerve blocks have become increasingly adopted in many centres and provide without doubt effect analgesia during the duration of the block and may be used also for intraoperative anaesthesia with or without sedation [22,23]. It may replace the need for general anaesthesia. So called walking spinal [24], infraclavicular block for low arm surgery [25] or the paravertable blocks for breast surgery are just some examples.

Liu *et al.* [26] conducted a meta-analysis published in 2005. Only major conduction blocks were considered to be regional anaesthesia. Regional anaesthesia was further separated into central neuraxial block and peripheral nerve block. Fifteen (1,003 patients) and seven (359 patients) trials for central neuraxial block and peripheral nerve block were included in the meta-analysis. Both central neuraxial block and peripheral nerve block were associated with increased induction time, reduced pain scores, and decreased need for postanaesthesia care unit analgesics. However, central neuraxial block was not associated with decreased postanaesthesia care unit bypass or time or reduced nausea despite reduced analgesics, and it was associated with a 35-min increase in total ambulatory surgery unit time. In contrast, peripheral nerve block was associated with decreased postanesthesia care unit need and decreased nausea but, again, not with decreased ambulatory surgery unit time. This meta-analysis indicates potential advantages for regional anaesthesia, such as decreased postanaesthesia care unit use, nausea, and postoperative pain. Although these factors have been proposed to reduce ambulatory surgery unit stay, neither central neuraxial block nor peripheral nerve block were associated with reduced ambulatory surgery unit time.

There are two more recent meta-analysis with a focus on the effects of paravertable block. Tahiri *et al.* [27] reviewed available studies comparing thoracic paravertebral block (TPVB) to general anaesthesia (GA) for ambulatory breast surgery. They found that was used instead of GA, pain scores were significantly decreased at 1 and 6 h postoperatively (mean difference of 2.48 (95%confidence interval (CI): 2.20–2.75) and 1.71 (95%CI: 1.64–1.78), respectively). Furthermore, postoperative analgesic consumption was significantly lower in patients who received TPVB compared with GA (relative risk (RR) 0.23, (95%CI: 0.15–0.37)). TPVB was also associated with significantly less postoperative nausea and vomiting (RR 0.27 (95%CI: 0.12–0.61)). Increased patient satisfaction and a shorter hospital stay also favoured TPVB over GA. Similar result was found in reviews by Thavaneswaran *et al.* [28] and Schnabel *et al.* [29].

There is a recent meta-analysis by De Oliviera *et al.* [30] around the effects of Transversus Abdominal Plane (TAP) blocks on pain course following laparoscopic surgery. It shows a significant effect of TAP block in the reduction of postoperative pain outcomes for laparoscopic surgical procedures. TAP block reduced early pain at rest, late pain at rest, and postoperative opioid consumption.

It seems reasonable to conclud that nerve blocks do provide effective analgesia and reduced need for rescue analgesia during the duration of its effect. Several adjuncts have been suggested in order to prolonged single block technique. Choi *et al.* [31] conducted a meta-analysis showing beneficial prolongation for dexamethasone. Dexamethasone prolonged the analgesic duration for long-acting local anaesthetic from 730 to 1306 min [mean difference 576 min, 95% confidence interval (CI) 522–631] and for intermediate from 168 to 343 min (mean 175, 95% CI 73–277). Motor block was prolonged from 664 to 1102 min (mean 438, 95% CI 89–787). The most recent trial demonstrated equivalent prolongation with perineural or systemic administration of dexamethasone compared with placebo.

Abdallah *et al.* [32] reviewed current public domain literature on the effects of local anaesthesia supplementation with dexmedetomidine for peripheral block. They found that dexmedetomidine is a potential LA adjuvant that can exhibit a facilitatory effect when administered intrathecally as part of spinal anaesthesia or peripherally as part of a brachial plexus block.

There is an increasing interest in ultra-sound guided blocks. The possibility to objectively view the needle placement is an interesting option. Schnabel *et al.* [33] published in the October 2013 issue of *BJA* and meta-analysis that show that there is evidence that ultra sound guided peripheral nerve catheters show a higher success rate and a lower risk for an accidental vascular puncture compared with nerve stimulated guidance. However, this difference resulted only in marginally lower postoperative pain scores at rest.

Lidocaine may have effects beyond the classical nerve blocking. It has been shown that systemic lidocaine posses clinical effects, reducing pain and discomfort. McCarthy *et al.* [34] conducted in 2010 a meta-analysis. They concluded that intravenous lidocaine infusion in the perioperative period is safe and has clear advantages in patients undergoing abdominal surgery. Patients receiving lidocaine infusion had lower pain scores, reduced postoperative analgesic requirements and decreased intraoperative anaesthetic requirements, as well as faster return of bowel function and decreased length of hospital stay.

## 6. Adjuncts Facilitating the Analgesic Effects

### 6.1. Dexamethasone

Single intra venous 0.1 mg per kilogram preoperative dose of dexamethasone has been shown to have combined anti-emetic and enhance analgesia [35]. De Olivera *et al.* [36] concluded from a meta-analysis published in 2011 that dexamethasone at doses more than 0.1 mg/kg is an effective adjunct in multimodal strategies to reduce postoperative pain and opioid consumption after surgery. The preoperative administration of the drug produces less variation of effects on pain outcomes. Halderon *et al.* [37] conducted a meta-analysis published in *BJA* in 2013. It concluded that a single i.v. perioperative dose of dexamethasone had small but statistically significant analgesic benefits. Forty-five studies involving 5,796 patients receiving dexamethasone 1.25–20 mg were included. Patients receiving dexamethasone had lower pain scores at 2 h {mean difference (MD) −0.49 [95% confidence interval (CI): −0.83, −0.15]} and 24 h [MD −0.48 (95% CI: −0.62, −0.35)] after surgery. Dexamethasone-treated patients used less opioids at 2 h [MD −0.87 mg morphine equivalents (95% CI: −1.40 to −0.33)] and 24 h [MD −2.33 mg morphine equivalents (95% CI: −4.39, −0.26)], required less rescue analgesia for intolerable pain [relative risk 0.80 (95% CI: 0.69, 0.93)], had longer time to first dose of analgesic [MD 12.06 min (95% CI: 0.80, 23.32)], and shorter stays in the post-anaesthesia care unit [MD −5.32 min (95% CI: −10.49 to −0.15)]. There was no dose-response with regard to the opioid-sparing effect. There was no increase in infection or delayed wound healing with dexamethasone, but blood glucose levels were higher at 24 h [MD 0.39 mmol L^−1^ (95% CI: 0.04, 0.74)]. Higher dose, 0.2 mg may however increase the risk for postoperative cognitive dysfunction as suggested in a study by Fang *et al.* [38].

### 6.2. Alfa-2-agonists

#### Clonidine and Dexmedetomidine

Both clonidine and dexmedetomidine has been studied as adjutants to paediatric general anaesthesia in order to reduce and ameliorate emergence agitation. A meta-analysis by Dahmani *et al.* [39] from 2010 showed that premedication with clonidine is superior to midazolam in producing sedation, decreasing post-operative pain and emergence agitation. However, the superiority of clonidine for PONV prevention remains unclear while other factors such as nausea prevention might interfere with this result. Bharti *et al.* [40] found in a recent study that clonidine 3 μg kg^−1^ provided effective postoperative analgesia and reduced morphine requirement when administered intravenously or in wound infiltration with bupivacaine. However, the incidence of complications was less with wound infiltration. Patients receiving i.v. clonidine had more hypotension (*p* < 0.01) and sedation (*p* < 0.001) compared with other groups.

He *et al.* [41] found in a systematic review that intraoperative use of dexmedetomidine was as effective as opioids in preventing postoperative pain and emergence agitation in children who had undergone tonsillectomy and adenoidectomy.

Dexmedetomidine has been studies not only as an adjunct to nerve block but as an alternative to propofol for sedation. Na *et al.* [42] studied outpatients undergoing cataract surgery. Dexmedetomidine was administered in group D at 0.6 µg/kg/h, and propofol and alfentanil was infused concomitantly in group P at a rate of 2 mg/kg/h and 20 µg/kg/h respectively. Sedation was titrated at Ramsay sedation score 3. They found postoperative that patients self-rating of satisfaction with sedation was 50.3 (6.2) in group D and 42.7 (8.7) in group P, which was statistically significant (*p* < 0.001). Systolic blood pressure was significantly lower in group D compared with group P from the beginning of the operation. HR, RR, and SpO(2) were comparable between the two groups. There were 8 cases (25.8%) of hypertension in group P, and 1 case (3.2%) in group D (*p* < 0.05). In contrast, 1 case (3.2%) of hypotension and 1 case (3.2%) of bradycardia occurred in group D. Whether the favourable effects seen from the use of dexmedetomidine as a complement to anaesthesia for cardiac surgery can be translated into day surgery for the elderly needs much further studies. Ji *et al.* [43] recently showed that perioperative dexmedetomidine use was associated with a decrease in postoperative mortality up to 1 year and decreased incidence of postoperative complications and delirium in patients undergoing cardiac surgery. The potential clinical impact of the decrease in systemic inflammatory response associated to dexmedetomidine administered during cholecystectomy needs also further studies. Kang *et al.* [44] found showed that patients that received following induction of anaesthesia a loading dose of dexmedetomidine (1.0 μg/kg), followed by infusion of dexmedetomidine at 0.5 μg/kg/h had significantly less inflammatory response following surgery as compared to saline-treated controls. 

There is a meta-analysis evaluating the addition of α-2-agonists on postopetive pain not explicitly following day surgery by Blaudszun *et al.* [45]. This review concluded that perioperative systemic α-2-agonists decrease postoperative opioid consumption, pain intensity, and nausea. Recovery times are not prolonged. Common adverse effects are bradycardia and arterial hypotension. The impact of these agents on chronic pain or hyperalgesia remains unclear because valid data are lacking.

### 6.3. Ketamine

Tawfic [46] made a review around ketamine and summarised that it has gained interest in patients with opioid tolerance, acute hyperalgesia, and neuropathic pain. It also has a role in the management of chronic pain including both cancer and noncancer pain. Its role in multi-modal analgesia following ambulatory surgery is however seemingly minor. Elia and Tramer [47] conducted a meta-analysis in 2005 and concluded that despite many published randomised trials, the role of ketamine, as a component of perioperative analgesia, remains unclear.

### 6.4. Gabapentin and Pregabalin

Peng *et al.* [48] did not find any benefit from the addition of 50 or 75 mg pregabalin as supplementary part of multi-modal pain management following cholecystectomy. On the day of surgery, patients received standard premedications as per our institution protocol: naprosyn 500 mg and acetaminophen 1,000 mg 1 h before surgery in the preadmission unit. Patients received standardized general anaesthesia. Induction of general anaesthesia was achieved with i.v. propofol 1–2 mg/kg and fentanyl 2–5 mg/kg. The surgeon administered local anaesthetic (bupivacaine 0.25% with epinephrine 1 in 200,000 to a volume of 30 mL) around the gall bladder bed and the laparoscopy port sites (10 mL of the same solution). Patients took their pregabalin medication or oral placebo 12 and 24 h after the first dose regardless of the level of pain they were experiencing. Patients who experienced insufficient pain relief were allowed to take supplementary combination tablets of acetaminophen 325 mg and codeine 30 mg 1–2 tablets orally every 4–6 h as needed (maximum of 12 tablets per day). Thus all patients followed the classic multi-modal pain strategy. Pegabalin in a dose of 150 mg administered preoperatively and further twice daily has been shown to have beneficial effects on pain following cholecystectomy. Balaban *et al.* [49] found 150 and 300 mg single dose Pregabalin administered prior to surgery to decrease pain scores and postoperative fentanyl consumption in patients after laparoscopic cholecystectomy in a dose-dependent manner, without any differences between the groups in side effects. Bekawi *et al.* [50] studied 150 mg pregabalin capsules administered two hours preoperative, 12 h postoperative and twice daily for 2 days. They found that the 24 hour-pethidine consumption was significantly lower (*p* < 0.001) in Pregabalin *versus* control. Pregabalin was also associated to significantly less (*p* < 0.001) patients with postoperative nausea, vomiting, sedation and dizziness *versus* control. Overall patient satisfaction with pain management was significantly higher (*p* < 0.001) in the pregabalin group. Sarakatsianou *et al.* [51] found likewise a reduced pain score, morphine consumption but a higher incidence of dizziness associated to a 300 mg dose provided the night before surgery and 1 h preoperatively. Yu *et al.* [52] showed in a meta-analysis that gabapentin as well as pregabalib were significantly more effective than place for the management of pain after spine surgery.

There is rather sparse data around the use, benefits from gabapentin as part of the pain management after ambulatory surgery. Kazak *et al.* [53] found beneficial effects from the addition of gabapentin 600 mg in conjunction with monitored anaesthesia care for nose surgery. Sen *et al.* [54] found a single-dose 1.2 g oral gabapentin 1 h before surgery to decreases the intensity of acute postoperative pain, tramadol consumption and the incidence and intensity of pain in the first 6 months after inguinal herniorrhaphy.

Duari *et al.* [55] conducted a meta-analysis of studies evaluating the effects of pregabalin and gabapentin. They concluded that gabapentin and pregabalin reduce pain and opioid consumption after surgery in confront with placebo, but comparisons with other standard post-operative regimens are not sufficient. Gabapentin and pregabalin seem not to have any influence on the prevention of PONV.

### 6.5. More Experimental

There are also other drugs, substances, that have been studied showing potential positive effects. Magnesium sulphate (MgSO_4_ 50 mg/kg in 100 mL of normal saline over 15 min before anesthesia induction, followed by an infusion of 15 mg/kg/h) has been shown by De Oliveiera *et al.* [56] to have positive effects. The authors concluded that systemic magnesium improves postoperative quality of recovery in patients undergoing outpatient segmental mastectomy. Systemic magnesium is a safe, inexpensive, efficacious strategy to improve quality of recovery after ambulatory surgery. Nitroglycerine ointment has been shown to have positive effects reducing pain after hemorroidectomy [57].

## 7. Discussion

The concept of multi-modal analgesia was shown effective for cholecystectomi already in 1996 by the Chung group in Toronto [3]. Patients were randomized to a treatment (n = 24) or control (n = 25) group and studied using a prospective, double-blind design. Preoperatively, at 45 min before induction of anesthesia, the treatment group received an intramuscular (IM) bolus injection of meperidine 0.6 mg/kg and ketorolac 0.5 mg/kg. The control group received two bolus IM injections of placebo (normal saline). Ten minutes before incision, local anesthesia (treatment group) or saline (control group) was infiltrated into the skin of each patient. Anesthetic management, postoperative pain, and nausea treatment were standardized. Pain and nausea assessment were done 1 h preoperatively, 0, 0.5, 1, 2, 3, and 4 h postoperatively, at discharge, and 10, 24, and 48 h postoperatively. Postoperatively, significantly more patients in the treatment group were without pain on arrival in the postanaesthesia care unit (PACU), 12/21 (57.1%) *vs.* 1/24 (4.2%) in the control group (*p* < 0.001). Similarly, the severity of pain was six-fold less in the treatment group than in the control group. The incidence of nausea in the PACU was significantly less in the treatment group; 4.7% *vs.* 29.5% in the control group (*p* < 0.05). Patients from the treatment group satisfied Postanaesthesia Discharge Score significantly earlier than those in the control group (281 +/− 12 min *vs.* 375 +/− 19 min; *p* < 005). They concluded that concomitant use of local anaesthetic, NSAID and opioid drugs proved to be highly effective in our patients, resulting in faster recovery and discharge. One of the goals is to reduce opioid related side effects and subsequently facilitate adequate pain relief but with a minimum of side effects. Zhao *et al.* [58] from the Chung group showed that the additional need for any morphine dose caused patient distress following ambulatory surgery; Once daily Morphine Equivalent Dose reaches a threshold, every 3–4 mg increase will be associated with 1 additional clinically meaningful opioid-related symptom, or 1 additional patient-day with an opioid-related Clinically Meaningful Event on the predefined opioid-related Symptom Distress Scale (SDS) questionnaire. The Kehlet group [59] one of the strongest promoters of opioid sparing postoperative analgesia tested a preventive therapy following breast surgery. The prevention regimen included a package consisting of preoperative paracetamol, dextromethorphan, celecoxib, gabapentin, dexamethasone, total intravenous anaesthesia and intraoperative ondansetron. The patients were prospectively scored according to PONV, pain during rest and mobilization and major side effects. Of 200 consecutive breast cancer patients, 191 received the full package. During the first 36 postoperative hours, 79.1% reported no PONV at all and only 3.7% reported severe PONV. At rest, 69.6% reported no or light pain and 3.1% reported severe pain, with corresponding values of 59.7% and 8.9% during arm mobilization. Mean postoperative morphine consumption was 2.2 mg. The only significant side effect was transient dizziness.

There is still no strong evidence for a pre-emptive effect from preoperative start of analgesia administration in clinical practice. Providing pain relief in order to secure adequate concentrations, effect after surgery, preventing pain to become severe is sound. Thus starting administration of paracetamol and an oral NSAID as premed has subsequently become well-accepted practice in many day surgical programs [60]. The aim of an analgesic technique should be not only to lower the pain scores but also to facilitate earlier mobilization and reduce perioperative complications [61].

Aluri *et al.* [62] conducted an Obstetric Anaesthetists’ Association approved electronic survey of all the UK lead obstetric anaesthetists between March and May 2013. They had a response rate of 81% was achieved with 96% of those who responded supporting the concept of enhanced recovery. Only 4% of units routinely discharged their patients on day one. There were a number of practices consistent with enhanced recovery. Postoperative pain was controlled by regular paracetamol (97%) and non-steroidal anti-inflammatory drugs (100% when not contraindicated), with oral opioids (68%) being used for breakthrough pain.

Multi modal analgesia has also been shown safe and effective for the management of pain following major surgery. Rafiq *et al.* [63] studied if an opiate sparing multimodal regimen consisting of dexamethasone, gabapentin, ibuprofen and paracetamol had better analgesic effect, less side effects and was safe compared to a traditional morphine and paracetamol regimen after cardiac surgery. Patients in the multimodal group demonstrated significantly lower average pain scores from the day of surgery throughout the third postoperative day. Extensive nausea and vomiting, was found in no patient in the multimodal group but in 13 patients in the morphine group, *p* < 0.001. Postoperative rise in individual creatinine levels demonstrated a non-significant rise in the multimodal group, 33.0 ± 53.4 *vs.* 19.9 ± 48.5, *p* = 0.133. Patients in the multimodal group suffered less major in-hospital events in crude numbers: myocardial infarction (MI) (1 *vs.* 2, *p* = 0.54), stroke (0 *vs.* 3, *p* = 0.075), dialysis (1 *vs.* 2, *p* = 0.54), and gastrointestinal (GI) bleeding (0 *vs.* 1, *p* = 0.31). 30-day mortality was 1 *vs.* 2, *p* = 0.54. The authors conclude that in patients undergoing cardiac surgery, a multimodal regimen offered significantly better analgesia than a traditional opiate regimen. Nausea and vomiting complaints were significantly reduced. No safety issues were observed with the multimodal regimen.

Although the classical papers by Eriksson *et al*. [2] and Michaloliakou et al [3] suggest benefits improved recovery a recent Cochrane Systematic Review by Gurusamy *et al.* [64] around pharmacological interventions for prevention or treatment of postoperative pain in people undergoing laparoscopic cholecystectomy states that there is a need for more high quality studies. A modest reduction in pain was found however effects on the proportion of participants who were discharged as day-surgery, the length of hospital stay, or the time taken to return to work were imprecise in all the comparisons in which these outcomes were reported (very low quality evidence). There was no mortality in any of the groups in the two trials that reported mortality (183 participants, very low quality evidence). Differences in serious morbidity outcomes between the groups were imprecise across all the comparisons (very low quality evidence). None of the trials reported patient quality of life or time taken to return to normal activity. Still the benefits from a procedural specific multi-modal opioid sparing analgesic approach seems reasonably well supported as presented by the Postoperative pain Org [65].

In conclusion, a multi-modal procedure specific analgesic strategy facilitates the postoperative course following day surgery; improves quality of care, reduce experience of PONV and shorten time to discharge. The exact impact of multi-modal—balanced analgesia on resumption of daily living and quality of life is not well-documented. Providing paracetamol in standard dose, adding an NSAID when feasible and not contra-indicated in lowest effective dose fort short period [66] and further providing an oral opioid for rescue medication is base therapy. The use of local anaesthesia in conjunction to surgery as infiltration, peripheral or central block provide several benefits.

## References

[1] Kehlet H., Dahl J.B. (1993). The value of “multimodal” or “balanced analgesia” in postoperative pain treatment. Anesth. Analg..

[2] Eriksson H., Tenhunen A., Korttila K. (1996). Balanced analgesia improves recovery and outcome after outpatient tubal ligation. Acta Anaesthesiol. Scand..

[3] Michaloliakou C., Chung F., Sharma S. (1996). Preoperative multimodal analgesia facilitates recovery after ambulatory laparoscopic cholecystectomy. Anesth. Analg..

[4] Bandolier. http://www.medicine.ox.ac.uk/bandolier/booth/painpag/Acutrev/Analgesics/Leagtab.html.

[5] Graham G.G., Davies M.J., Day R.O., Mohamudally A., Scott K.F. (2013). The modern pharmacology of paracetamol: Therapeutic actions, mechanism of action, metabolism, toxicity and recent pharmacological findings. Inflammopharmacology.

[6] Blieden M., Paramore L.C., Shah D., Ben-Joseph R. (2014). A perspective on the epidemiology of acetaminophen exposure and toxicity in the United States. Expert Rev. Clin. Pharmacol..

[7] FDA. http://www.fda.gov/Drugs/DrugSafety/ucm381644.htm.

[8] Qi D.S., May L.G., Zimmerman B., Peng P., Atillasoy E., Brown J.D., Cooper S.A. (2012). A randomized, double-blind, placebo-controlled study of acetaminophen 1000 mg *versus* acetaminophen 650 mg for the treatment of postsurgical dental pain. Clin. Ther..

[9] Yue Y., Collaku A., Brown J., Buchanan W.L., Reed K., Cooper S.A., Otto J. (2013). Efficacy and speed of onset of pain relief of fast-dissolving paracetamol on postsurgical dental pain: two randomized, single-dose, double-blind, placebo-controlled clinical studies. Clin Ther..

[10] Holmér Pettersson P., Owall A., Jakobsson J. (2004). Early bioavailability of paracetamol after oral or intravenous administration. Acta Anaesthesiol. Scand..

[11] Brett C.N., Barnett S.G., Pearson J. (2012). Postoperative plasma paracetamol levels following oral or intravenous paracetamol administration: a double-blind randomised controlled trial. Anaesth. Intensive Care.

[12] McNicol E.D., Tzortzopoulou A., Cepeda M.S., Francia M.B., Farhat T., Schumann R. (2011). Single-dose intravenous paracetamol or propacetamol for prevention or treatment of postoperative pain: a systematic review and meta-analysis. Br. J. Anaesth..

[13] Apfel C.C., Souza K., Portillo J., Dalal P., Bergese S.D. (2014). Patient Satisfaction with Intravenous Acetaminophen: A Pooled Analysis of Five Randomized, Placebo-Controlled Studies in the Acute Postoperative Setting. J. Healthc Qual..

[14] Bailey E., Worthington H.V., van Wijk A., Yates J.M., Coulthard P., Afzal Z. (2013). Ibuprofen and/or paracetamol (acetaminophen) for pain relief after surgical removal of lower wisdom teeth. Cochrane Database Syst Rev..

[15] Jones P., Lamdin R. (2010). Oral cyclo-oxygenase 2 inhibitors *versus* other oral analgesics for acute soft tissue injury: systematic review and meta-analysis. Clin. Drug Investig..

[16] Kawano T., Takahashi T., Iwata H., Morikawa A., Imori S., Waki S., Tamura T., Yamazaki F., Eguchi S., Kumagai N., Yokoyama M. (2014). Effects of ketoprofen for prevention of postoperative cognitive dysfunction in aged rats. J. Anesth..

[17] Cai W.X., Ma L., Zheng L.W., Kruse-Gujer A., Stübinger S., Lang N.P., Zwahlen R.A. (2014). Influence of non-steroidal anti-inflammatory drugs (NSAIDs) on osseointegration of dental implants in rabbit calvaria. Clin. Oral Implants Res..

[18] Clarke R., Derry S., Moore R.A. (2012). Single dose oral etoricoxib for acute postoperative pain in adults. Cochrane Database Syst Rev..

[19] Singh B.K., Haque S.E., Pillai K.K. (2014). Assessment of nonsteroidal anti-inflammatory drug-induced cardiotoxicity. Expert Opin. Drug Metab. Toxicol..

[20] Wickerts L., Warrén Stomberg M., Brattwall M., Jakobsson J. (2011). Coxibs: Is there a benefit when compared to traditional non-selective NSAIDs in postoperative pain management?. Minerva Anestesiol..

[21] Derry C.J., Derry S., Moore R.A. (2013). Single dose oral ibuprofen plus paracetamol (acetaminophen) for acute postoperative pain. Cochrane Database Syst. Rev..

[22] O’Donnell B.D., Iohom G. (2008). Regional anesthesia techniques for ambulatory orthopedic surgery. Curr. Opin. Anaesthesiol..

[23] Moore J.G., Ross S.M., Williams B.A. (2013). Regional anesthesia and ambulatory surgery. Curr. Opin. Anaesthesiol..

[24] Nair G.S., Abrishami A., Lermitte J., Chung F. (2009). Systematic review of spinal anaesthesia using bupivacaine for ambulatory knee arthroscopy. Br. J. Anaesth..

[25] Chin K.J., Alakkad H., Adhikary S.D., Singh M. (2013). Infraclavicular brachial plexus block for regional anaesthesia of the lower arm. Cochrane Database Syst. Rev..

[26] Liu S.S., Strodtbeck W.M., Richman J.M., Wu C.L. (2005). A comparison of regional *versus* general anesthesia for ambulatory anesthesia: A meta-analysis of randomized controlled trials. Anesth. Analg..

[27] Tahiri Y., Tran de Q.H., Bouteaud J., Xu L., Lalonde D., Luc M., Nikolis A. (2011). General anaesthesia *versus* thoracic paravertebral block for breast surgery: A meta-analysis. J. Plast Reconstr. Aesthet. Surg..

[28] Thavaneswaran P., Rudkin G.E., Cooter R.D., Moyes D.G., Perera C.L., Maddern G.J. (2010). Brief reports: Paravertebral block for anesthesia: A systematic review. Anesth. Analg..

[29] Schnabel A., Reichl S.U., Kranke P., Pogatzki-Zahn E.M., Zahn P.K. (2010). Efficacy and safety of paravertebral blocks in breast surgery: A meta-analysis of randomized controlled trials. Br. J. Anaesth..

[30] De Oliveira G.S., Castro-Alves L.J., Nader A., Kendall M.C., McCarthy R.J. (2014). Transversus abdominis plane block to ameliorate postoperative pain outcomes after laparoscopic surgery: A meta-analysis of randomized controlled trials. Anesth. Analg..

[31] Choi S., Rodseth R., McCartney C.J. (2014). Effects of dexamethasone as a local anaesthetic adjuvant for brachial plexus block: A systematic review and meta-analysis of randomized trials. Br. J. Anaesth..

[32] Abdallah F.W., Brull R. (2013). Facilitatory effects of perineural dexmedetomidine on neuraxial and peripheral nerve block: A systematic review and meta-analysis. Br. J. Anaesth..

[33] Schnabel A., Meyer-Frießem C.H., Zahn P.K., Pogatzki-Zahn E.M. (2013). Ultrasound compared with nerve stimulation guidance for peripheral nerve catheter placement: A meta-analysis of randomized controlled trials. Br. J. Anaesth..

[34] McCarthy G.C., Megalla S.A., Habib A.S. (2010). Impact of intravenous lidocaine infusion on postoperative analgesia and recovery from surgery: A systematic review of randomized controlled trials. Drugs.

[35] Jakobsson J. (2010). Preoperative single-dose intravenous dexamethasone during ambulatory surgery: Update around the benefit *versus* risk. Curr. Opin. Anaesthesiol..

[36] De Oliveira G.S., Almeida M.D., Benzon H.T., McCarthy R.J. (2011). Perioperative single dose systemic dexamethasone for postoperative pain: A meta-analysis of randomized controlled trials. Anesthesiology.

[37] Waldron N.H., Jones C.A., Gan T.J., Allen T.K., Habib A.S. (2013). Impact of perioperative dexamethasone on postoperative analgesia and side-effects: Systematic review and meta-analysis. Br. J. Anaesth..

[38] Fang Q., Qian X., An J., Wen H., Cope D.K., Williams J.P. (2014). Higher Dose Dexamethasone Increases Early Postoperative Cognitive Dysfunction. J. Neurosurg. Anesthesiol..

[39] Dahmani S., Brasher C., Stany I., Golmard J., Skhiri A., Bruneau B., Nivoche Y., Constant I., Murat I. (2010). Premedication with clonidine is superior to benzodiazepines. A meta analysis of published studies. Acta Anaesthesiol. Scand..

[40] harti N., Dontukurthy S., Bala I., Singh G. (2013). Postoperative analgesic effect of intravenous (i.v.) clonidine compared with clonidine administration in wound infiltration for open cholecystectomy. Br. J. Anaesth..

[41] He X.Y., Cao J.P., Shi X.Y., Zhang H. (2013). Dexmedetomidine *versus* morphine or fentanyl in the management of children after tonsillectomy and adenoidectomy: A meta-analysis of randomized controlled trials. Ann. Otol. Rhinol. Laryngol..

[42] Na H.S., Song I.A., Park H.S., Hwang J.W., Do S.H., Kim C.S. (2011). Dexmedetomidine is effective for monitored anesthesia care in outpatients undergoing cataract surgery. Korean J. Anesthesiol..

[43] Ji F., Li Z., Nguyen H., Young N., Shi P., Fleming N., Liu H. (2013). Perioperative dexmedetomidine improves outcomes of cardiac surgery. Circulation.

[44] Kang S.H., Kim Y.S., Hong T.H., Chae M.S., Cho M.L., Her Y.M., Lee J. (2013). Effects of dexmedetomidine on inflammatory responses in patients undergoing laparoscopic cholecystectomy. Acta Anaesthesiol. Scand..

[45] Blaudszun G., Lysakowski C., Elia N., Tramèr M.R. (2012). Effect of perioperative systemic α2 agonists on postoperative morphine consumption and pain intensity: Systematic review and meta-analysis of randomized controlled trials. Anesthesiology.

[46] Tawfic Q.A. (2013). A review of the use of ketamine in pain management. J. Opioid Manag..

[47] Elia N., Tramèr M.R. (2005). Ketamine and postoperative pain—A quantitative systematic review of randomised trials. Pain.

[48] Peng P.W., Li C., Farcas E., Haley A., Wong W., Bender J., Chung F. (2010). Use of low-dose pregabalin in patients undergoing laparoscopic cholecystectomy. Br. J. Anaesth..

[49] Balaban F., Yağar S., Özgök A., Koç M., Güllapoğlu H. (2012). A randomized, placebo-controlled study of pregabalin for postoperative pain intensity after laparoscopic cholecystectomy. J. Clin. Anesth..

[50] Bekawi M.S., El Wakeel L.M., Al Taher W.M., Abdel Mageed W.M. (2014). Clinical Study Evaluating Pregabalin Efficacy and Tolerability for Pain Management in Patients Undergoing Laparoscopic Cholecystectomy. Clin. J. Pain..

[51] Sarakatsianou C., Theodorou E., Georgopoulou S., Stamatiou G., Tzovaras G. (2013). Effect of pre-emptive pregabalin on pain intensity and postoperative morphine consumption after laparoscopic cholecystectomy. Surg. Endosc..

[52] Yu L., Ran B., Li M., Shi Z. (2013). Gabapentin and pregabalin in the management of postoperative pain after lumbar spinal surgery: A systematic review and meta-analysis. Spine.

[53] Kazak Z., Meltem Mortimer N., Sekerci S. (2010). Single dose of preoperative analgesia with gabapentin (600 mg) is safe and effective in monitored anesthesia care for nasal surgery. Eur. Arch. Otorhinolaryngol..

[54] Sen H., Sizlan A., Yanarateş O., Senol M.G., Inangil G., Sücüllü I., Ozkan S., Dağli G. (2009). The effects of gabapentin on acute and chronic pain after inguinal herniorrhaphy. Eur. J. Anaesthesiol..

[55] Dauri M., Faria S., Gatti A., Celidonio L., Carpenedo R., Sabato A.F. (2009). Gabapentin and pregabalin for the acute post-operative pain management. A systematic-narrative review of the recent clinical evidences. Curr. Drug Targets.

[56] De Oliveira G.S., Bialek J., Fitzgerald P., Kim J.Y., McCarthy R.J. (2013). Systemic magnesium to improve quality of post-surgical recovery in outpatient segmental mastectomy: A randomized, double-blind, placebo-controlled trial. Magnes. Res..

[57] Karanlik H., Akturk R., Camlica H., Asoglu O. (2009). The effect of glyceryl trinitrate ointment on posthemorrhoidectomy pain and wound healing: Results of a randomized, double-blind, placebo-controlled study. Dis. Colon. Rectum..

[58] Zhao S.Z., Chung F., Hanna D.B., Raymundo A.L., Cheung R.Y., Chen C. (2004). Dose-response relationship between opioid use and adverse effects after ambulatory surgery. J. Pain Symptom Manage.

[59] Gärtner R., Kroman N., Callesen T., Kehlet H. (2010). Multimodal prevention of pain, nausea and vomiting after breast cancer surgery. Minerva Anestesiol..

[60] O’Hanlon D.M., Thambipillai T., Colbert S.T., Keane P.W., Given H.F. (2001). Timing of pre-emptive tenoxicam is important for postoperative analgesia. Can. J. Anaesth..

[61] Joshi G.P. (1994). Postoperative pain management. Int. Anesthesiol. Clin..

[62] Aluri S., Wrench I.J. (2014). Enhanced recovery from obstetric surgery: A UK survey of practice. Int. J. Obstet. Anesth..

[63] Rafiq S., Steinbrüchel D.A., Wanscher M.J., Andersen L.W., Navne A., Lilleoer N.B., Olsen P.S. (2014). Multimodal analgesia *versus* traditional opiate based analgesia after cardiac surgery, a randomized controlled trial. J. Cardiothorac. Surg..

[64] Gurusamy K.S., Vaughan J., Toon C.D., Davidson B.R. (2014). Pharmacological interventions for prevention or treatment of postoperative pain in people undergoing laparoscopic cholecystectomy. Cochrane Database Syst. Rev..

[65] Homepage of Prospect. http://www.postoppain.org/frameset.htm.

[66] Bhala N., Emberson J., Merhi A., Abramson S., Arber N., Baron J.A., Bombardier C., Cannon C., Farkouh M.E., Coxib and traditional NSAID Trialists' (CNT) Collaboration (2013). Vascular and upper gastrointestinal effects of non-steroidal anti-inflammatory drugs: Meta-analyses of individual participant data from randomised trials. Lancet.

